# Comparison of Two New Robust Parameter Estimation Methods for the Power Function Distribution

**DOI:** 10.1371/journal.pone.0160692

**Published:** 2016-08-08

**Authors:** Muhammad Shakeel, Muhammad Ahsan ul Haq, Ijaz Hussain, Alaa Mohamd Abdulhamid, Muhammad Faisal

**Affiliations:** 1 Department of Statistics, Government Degree College for Boys Hujra Shah Muqeem, Okara, Pakistan; 2 College of Statistical and Actuarial Sciences, University of the Punjab, Lahore, Pakistan; 3 Department of Statistics, Quaid-i-Azam University, Islamabad, Pakistan; 4 Arriyadh Community College, King Saud University, Riyadh, Saudi Arabia; 5 Bradford Institute for Health Research, Bradford Teaching Hospitals, NHS Foundation Trust, Bradford, United Kingdom; University of East Piedmont, ITALY

## Abstract

Estimation of any probability distribution parameters is vital because imprecise and biased estimates can be misleading. In this study, we investigate a flexible power function distribution and introduced new two methods such as, probability weighted moments, and generalized probability weighted methods for its parameters. We compare their results with L-moments, trimmed L-moments by a simulation study and a real data example based on performance measures such as, mean square error and total deviation. We concluded that all the methods perform well in the case of large sample size (n>30), however, the generalized probability weighted moment method performs better for small sample size.

## Introduction

Power function distribution is a flexible and simple distribution that may helpful for modeling the failure data. It is often used in the assessment of semiconductor devices and electrical component reliability [1]. Zarrin et al. [2] applied power function distribution to assess component failure of semi-conductor device data by using both the maximum likelihood and Bayesian estimation methods. A brief discussion about this distribution and its various properties are described in [3]. Theoretically, power function distribution has an inverse relationship with the standard Pareto distribution, and it is also a special case of Pearson type I distribution [3]. The moments of the power function distribution are simply the negative moments of the Pareto distribution [4]. Moments of order statistics for power function distribution are calculated by [5]. Athar and Faizan [6] derived the explicit expressions for single and product moments. They also showed the recurrence relationship for single and product moments of lower generalized order statistics of power distribution function. Chang [7] described the characterizations of the power function distribution by means of the independence of record values. Saran and Pandey [8] considered the *k*^*th*^ record value for the parameter estimation of the power function distribution. Omar and Low [9] developed the Bayesian estimate for the shape parameter of the generalized power function distribution by considering both the informative and non-informative priors under mean square error loss function. Moreover, Sultan et al. [10] estimated the scale parameter of the power function distribution by using Bayesian method with three double types of priors and three single types of priors’ distributions. Bhatt [11] showed the characterization of power function distribution through expectation of non-constant function of a random variable. Lutful-Kabir and Ahsanullah [12] estimated the parameter of a Power function distribution by using linear function of the order statistics. Further, Haq et al. [13] developed the generalized form of Power function distribution that known as Transmuted Power Function distribution. Haq et al. [14] also commented on Kumaraswamy Power Function and suggested its suitable applications.

Zaka et al. [15] presented the modification of maximum likelihood, moments and percentile estimators of the two parameters power function distribution. Saleem et al. [16] derived the finite mixture density of power function distribution and they also derived the Bayesian estimators for censored and complete sample. Recently, Shahzad et al. [17] found that the L-moments method performs better than Trim L-moments method in case of power function distribution.

In this paper, we introduced two new parameter estimation methods such as, probability weighted moments and generalized probability weighted moments for two parameter power function distribution. We derive the expressions for parameter estimation of them. We compare the performance of these methods with the L moments and TL-moments methods by a simulation study and a real data example based on performance measures such as, mean square error and total deviation.

## The Power Function Distribution

We consider the probability density function (*pdf*) and cumulative distribution function (*cdf*) of the power function distribution:

$$f(x)=\frac{\alpha }{\theta^{\alpha }}x^{\alpha -1}\;0<x<\theta \;,\alpha >0,\theta >0$$ (1)

$$F(x)={\left(\frac{x}{\theta }\right)}^{\alpha }\;0<x<\theta \;,\alpha >0,\theta >0$$ (2)

Where *α* is shape parameter and *θ* is the scale parameter.

### L-Moments

The L-moments as an analogy to the conventional moments [18] and it can be defined as any random variable whose mean exists [19]. L-moments are estimated by linear combination of order statistics. They are robust to the outliers and the influence of sample variation [20]. L-moments are commonly considered as more efficient parameter estimation method than the maximum likelihood method particularly for small sample size.

If *X* is a continuous random variable with distribution function *F*(*x*) and *Q*(*x*) as a quantile function, then the L-moments of *r*^*th*^ order random variable are defined as:

$$\lambda_{r}=\frac{1}{r}\sum_{j=0}^{r-1}{\left(-1\right)}^{r}\;\left(\begin{array}{c} r-1\\ j \end{array}\right)\;E\left(X_{r-j:r}\right);\;\text{r}=1,2,3\ldots ..$$ (3)

and the expected value of *r*^*th*^ order statistics of a random sample of size *n* has the form

$$E\left(X_{r-j:r}\right)=\frac{n!}{\left(r-1\right)!\left(n-r\right)!}\int_{0}^{1}Q\left(F\right)F^{r-1}{\left(1-F\right)}^{n-r}dF$$ (4)

Let *x*_1_,*x*_2_,*x*_3_,…,*x*_*n*_ be a sample and *x*_(1)_ ≤ *x*_(2)_ ≤ *x*_(3)_ ≤ ….. ≤ *x*_(*n*)_ an ordered sample, then the *r*^*th*^ unbiased empirical L-moments are defined by Asquith [21] and can be written as;

$$l_{r}=\frac{1}{r}\sum_{i=1}^{n}\left[\frac{\sum_{j=0}^{r-1}{\left(-1\right)}^{j}\left(\begin{array}{c} r-1\\ j \end{array}\right)\left(\begin{array}{c} i-1\\ r-1-j \end{array}\right)\left(\begin{array}{c} n-i\\ j \end{array}\right)}{\left(\begin{array}{c} n\\ r \end{array}\right)}\right]X_{i:n}$$ (5)

L-moments of the power function distribution can be derived from Eq (3) i.e.

$$\lambda_{1}=\frac{\alpha \theta }{\alpha +1}$$

$$\lambda_{2}=\frac{\alpha \theta }{\left(\alpha +1)(2\alpha +1\right)}$$

The estimators of the power function parameters are *α* and *θ*. In order to obtain L-moments in terms of *l*_1_ and *l*_2_ by equating the λ_1_ to *l*_1_ and λ_2_ to *l*_2_, we get:

$$\hat{\alpha }=\frac{l_{1}-l_{2}}{{2l}_{2}}\;\mathrm{and}\;\hat{\theta }=\frac{l_{1}(l_{1}+l_{2})}{l_{1}-l_{2}}$$

### Trimmed L-moments

Elamir and Seheult [22] derived Trimmed L-moments (TL-moments) that is a natural generalization of L-moments because it does not need the mean of the underlying distribution to be exist e.g., Cauchy distribution [23]. Initially, TL-moments were developed as supplement for other methods particularly when dealing with outliers in the data [24]. In fact, the expected value of order statistics *E*(*X*_*r*−*j*:*r*_) is replaced by *E*(*X*_*r*+*t*1−*j*:*r*+*t*1+*t*2_) for large samples in L-moments where the increased size is the total amount of trimming. Thus the *r*^*th*^ order TL-moments are denoted as $\lambda_{r}^{\left(t1,t2\right)}$.

$$\lambda_{r}^{\left(t1,t2\right)}=\frac{1}{r}\sum_{j=0}^{r-1}{\left(-1\right)}^{r}\;\left(\begin{array}{c} r-1\\ j \end{array}\right)\;E\left(X_{r+t1-j:r+t1+t2}\right)\;r=1,2,\ldots$$ (6)

TL-moments reduce to L-moments, if we put *t*1 = *t*2 = 0 in Eq (6). Here, symmetric case of TL-moments is considered i.e., *t*1 = *t*2 = *t*. For the symmetric case Eq (6) can be rewritten as:

$$\lambda_{r}^{\left(t\right)}=\frac{1}{r}\sum_{j=0}^{r-1}{\left(-1\right)}^{r}\;\left(\begin{array}{c} r-1\\ j \end{array}\right)\;E\left(X_{r+t-j:r+2t}\right)\;r=1,2,\ldots$$ (7)

The unbiased TL-moments where sampled TL-moments equivalent to population TL-moments are defined by Asquith [21] as following:

$$l_{r}^{\left(t\right)}=\frac{1}{r}\sum_{i=r+t}^{n-t}\left[\frac{\sum_{j=0}^{r-1}{\left(-1\right)}^{j}\left(\begin{array}{c} r-1\\ j \end{array}\right)\left(\begin{array}{c} i-1\\ r+t-j-1 \end{array}\right)\left(\begin{array}{c} n-i\\ t+j \end{array}\right)}{\left(\begin{array}{c} n\\ r+2t \end{array}\right)}\right]x_{i:n}$$ (8)

The TL-moments of the power function distribution for *t* = 1 are:

$$\lambda_{1}^{\left(1\right)}=\frac{6\alpha^{2}\theta }{\left(2\alpha +1)(3\alpha +1\right)}$$

$$\lambda_{2}^{\left(1\right)}=\frac{6\alpha^{2}\theta }{\left(2\alpha +1)(3\alpha +1)(4\alpha +1\right)}$$

The estimators of the power function parameters *α* and *θ* by means of TL-moments can be obtained in terms of $l_{1}^{\left(1\right)}$ and $l_{2}^{\left(1\right)}$ by equating the $\lambda_{1}^{\left(1\right)}$ to $l_{1}^{\left(1\right)}$ and $\lambda_{2}^{\left(1\right)}$ to $l_{2}^{\left(1\right)}$:

$$\hat{\alpha }=\frac{l_{1}^{\left(1\right)}-l_{2}^{\left(1\right)}}{{4l}_{2}^{\left(1\right)}}$$

$$\hat{\theta }=\frac{l_{1}^{\left(1\right)}({3l}_{1}^{\left(1\right)}+l_{2}^{\left(1\right)})(l_{1}^{\left(1\right)}+l_{2}^{\left(1\right)})}{3{\left(l_{1}^{\left(1\right)}-l_{2}^{\left(1\right)}\right)}^{2}}$$

### Probability Weighted Moments

Greenwood et al. [25] proposed probability weighted moments (PWMs), that is the generalization of usual moments of the probability distribution. It is unbiased, stable and particularly attractive when the cumulative distribution function *F*_*X*_(*x*) of a distribution has a closed form [26]. It is commonly used for estimating the parameters of the distributions that are analytically expressible in quantile form such as, Wakeby and Tukey’s Lambda distribution [27]. If *X* is the random variable with *cdf F*_*X*_(*x*), then the PWM are expressed as:

$$M_{p,u,v}=E\left[X^{p}{\left\{F_{X}\left(x\right)\right\}}^{u}{\left\{\left(1-F_{X}\left(x\right)\right)\right\}}^{v}\right]$$ (9)

where *p*,*u*,*v* are integer numbers. If the inverse distribution function *Q*(*F*) can be written in closed form, then an alternative form of the PWM is devised as:

$$M_{p,u,v}=\int_{0}^{1}Q{\left(F\right)}^{p}F^{u}{\left(1-F\right)}^{v}dF.$$ (10)

If *u* = *v* = 0 and *p* is non-negative then *M*_*p*,0,0_ are the non-central conventional moments. Particularly useful special cases of PWM are *α*_*v*_ = *M*_1,0,*v*_ and *β*_*u*_ = *M*_1,*u*,0_.

Let *x*_(1)_, *x*_(2)_, *x*_(3)_,….,*x*_(*n*)_ be a random sample of size *n* from the distribution function *F*(*x*) and *x*_(1)_ < *x*_(2)_ < *x*_(3)_,….< *x*_(*n*)_ be the corresponding ordered sample. Landwehr et al. [28] proposed an unbiased estimator of PWM as:

$${\hat{\beta }}_{u}={\hat{M}}_{1,u,0}=n^{-1}\sum_{j=1}^{n}\frac{\left(j-1\right)\left(j-2\right)\ldots \left(j-u\right)}{\left(n-1\right)\left(n-2\right)\ldots \left(n-u\right)}X_{j:n}$$ (11)

The general expression of PWM is given in Eq (10).

The PWM for the power function distribution is derived as follow by using Eq (10):

$$\beta_{0}=M_{1,0,0}=\frac{\alpha \theta }{\alpha +1}\;\mathrm{and}\;\beta_{1}=M_{1,1,0}=\frac{\alpha \theta }{2\alpha +1}$$

The estimators of the power function parameters α and θ by means of PWM are obtained in terms of ${\hat{M}}_{1,0,0}$ and ${\hat{M}}_{1,1,0}$ by equating the *M*_1,0,0_ to ${\hat{M}}_{1,0,0}$ and *M*_1,1,0_ to ${\hat{M}}_{1,1,0}$:

$$\hat{\alpha }=\frac{{\hat{M}}_{1,0,0}-{\hat{M}}_{1,1,0}}{2{\hat{M}}_{1,1,0}-{\hat{M}}_{1,0,0}}$$

$$\hat{\theta }=\frac{{\hat{M}}_{1,0,0}{\hat{M}}_{1,1,0}}{{\hat{M}}_{1,0,0}-{\hat{M}}_{1,1,0}}$$

### Generalized Probability Weighted Moments

Rasmussen [29] proposed generalized probability weighted moments (GPWM) as an extension of PWM. It is used to estimate the parameters of such probability distributions that can be expressed in inverse form. The PWM only considers the non-negative integers on the exponent while GPWM method is unrestricted to the smallest non-negative integers on the exponent [30].

The common practice of GPWM of order *p* = 1 and *v* = 0 takes the following form

$$M_{1,u,0}=E[X^{1}{{\{F}_{X}(x)\}}^{u}]$$

The PWM involves consideration of *u* = 0 and *u* = 1 in the above equation for a two parametric distribution while GPWM method considers *u* = *u*_1_ and *u* = *u*_2_ where *u*_1_ and *u*_2_ are either to be small or non-negative integers. The empirical estimate of GPWM proposed by Hosking [31] is given as: ${\hat{M}}_{1,u,0}=\frac{1}{n}\sum_{i=1}^{n}x_{i}{\left(\frac{i-0.35}{n}\right)}^{u}$.

The estimated GPWM estimates for the power function distribution can be obtained as:

$$M_{1,u_{1},0}=\frac{\alpha \theta }{\alpha +\alpha u_{1}+1}$$

$$M_{1,u_{2},0}=\frac{\alpha \theta }{\alpha +\alpha u_{2}+1}$$

$$\hat{\alpha }=\frac{{\hat{M}}_{1,u_{1},0}-{\hat{M}}_{1,u_{2},0}}{{\hat{M}}_{1,u_{2,0}}(1+u_{2})-{\hat{M}}_{1,u_{1,0}}(1+u_{1})}$$

$$\hat{\theta }=\frac{{\hat{M}}_{1,u_{1},0}{\hat{M}}_{1,u_{2},0}(u_{2}-u_{1})}{{\hat{M}}_{1,u_{1},0}-{\hat{M}}_{1,u_{2},0}}$$

## A Simulation Study

Monte Carlo simulation is designed to investigate the sampling behaviour of the L-moments (LM), Trimmed L-moments (TLM), probability weighted moments (PWMs) and generalized probability weighted moments (GPWMs) estimators. This comparison is carried out by taking the sample of sizes (*n* = 10, 25, 50, 100, 150, 250, and 500). The accuracy of the estimates is compared by using following performance measures mean square error (MSE) and total deviation (TD). The *lmomco* package in R software by Asquith [32] is used for this analysis.

The results of our simulation study are presented in the Tables 1–4. We can assess the accuracy of these estimators in terms of bias, means square errors (MSE), and total deviation (TD). The results show that GPWM is relatively better (smaller MSE and TD) than LM, TLM, and PWM for small sample size and for all parameters values of *α* and *θ*. However, the bias of GPWM is slightly more than LM, TLM, and PWM for small sample size. Moreover, bias decreases as the sample size increases. The MSE for *α* and *θ* rise for higher parameters values.

**Table 1:** Comparison of the estimation methods for (α = 0.6, θ = 2.5).

n		${\hat{\alpha }}_{LM}$	${\hat{\theta }}_{LM}$	${\hat{\alpha }}_{TLM}$	${\hat{\theta }}_{TLM}$	${\hat{\alpha }}_{PWM}$	${\hat{\theta }}_{PWM}$	${\hat{\alpha }}_{GPWM}$	${\hat{\theta }}_{GPWM}$
10	Mean	0.60936	2.62931	0.64342	2.72773	0.61560	2.61683	0.63338	2.52208
	MSE	0.07474	0.29290	0.12522	0.87980	0.06977	0.28055	0.06396	0.19446
	T.D	0.067324		0.16346		0.07273		0.06447	
25	Mean	0.60168	2.53893	0.60769	2.57847	0.60087	2.53823	0.61112	2.51155
	MSE	0.02194	0.08229	0.02777	0.19562	0.02169	0.08199	0.02182	0.06449
	T.D	0.01838		0.04419		0.01675		0.02315	
50	Mean	0.60023	2.51947	0.60157	2.53020	0.60118	2.51832	0.60406	2.50839
	MSE	0.00994	0.03657	0.01202	0.08170	0.00999	0.03770	0.01056	0.03014
	T.D	0.00817		0.01469		0.00930		0.01012	
100	Mean	0.60048	2.51157	0.60107	2.51476	0.59829	2.50882	0.60214	2.50452
	MSE	0.00486	0.01775	0.00566	0.03812	0.00487	0.01714	0.00509	0.01454
	T.D	0.00543		0.00769		0.00567		0.00536	
150	Mean	0.60019	2.50639	0.60118	2.50916	0.59984	2.50549	0.60144	2.50384
	MSE	0.00321	0.01167	0.00382	0.02631	0.00308	0.01171	0.00336	0.00952
	T.D	0.00287		0.00564		0.00193		0.00393	
250	Mean	0.59986	2.50355	0.59973	2.50758	0.60008	2.50436	0.60109	2.50127
	MSE	0.00193	0.00689	0.00224	0.01523	0.00192	0.00684	0.00205	0.00565
	T.D	0.00119		0.00258		0.00187		0.00233	
500	Mean	0.60022	2.50169	0.59919	2.50359	0.59975	2.50225	0.59945	2.50130
	MSE	0.00094	0.00342	0.00109	0.00734	0.01093	0.00342	0.01090	0.00274
	T.D	0.00103		0.00008		0.00048		0.00040	

**Table 2:** Comparison of the estimation methods for (α = 0.7, θ = 3).

n		${\hat{\alpha }}_{LM}$	${\hat{\theta }}_{LM}$	${\hat{\alpha }}_{TLM}$	${\hat{\theta }}_{TLM}$	${\hat{\alpha }}_{PWM}$	${\hat{\theta }}_{PWM}$	${\hat{\alpha }}_{GPWM}$	${\hat{\theta }}_{GPWM}$
10	Mean	0.72263	3.11949	0.75321	3.22605	0.72133	3.1235	0.72462	3.04562
	MSE	0.10206	0.30453	0.15670	0.97342	0.09903	0.31992	0.07690	0.22856
	T.D	0.07216		0.15136		0.07164		0.05038	
25	Mean	0.70500	3.04684	0.71679	3.06086	0.70314	3.04925	0.70824	3.02145
	MSE	0.02852	0.09460	0.03898	0.21583	0.02840	0.09249	0.02857	0.07499
	T.D	0.02276		0.21954		0.02090		0.01892	
50	Mean	0.70350	3.01898	0.70525	3.03297	0.70099	3.02094	0.70200	3.01320
	MSE	0.01322	0.04319	0.01628	0.09559	0.01336	0.04331	0.01308	0.03492
	T.D	0.01133		0.09668		0.00839		0.00725	
100	Mean	0.70081	3.00838	0.70217	3.01333	0.70066	3.00911	0.70070	3.00641
	MSE	0.00629	0.02077	0.00763	0.04392	0.00650	0.02157	0.00670	0.01695
	T.D	0.00395		0.04409		0.00397		0.00314	
150	Mean	0.70017	3.00534	0.70086	3.01065	0.69991	3.00821	0.70138	3.00332
	MSE	0.00433	0.01331	0.00500	0.02879	0.00431	0.01351	0.00447	0.01082
	T.D	0.00202		0.02890		0.00261		0.00308	
250	Mean	0.70125	3.00445	0.70138	3.00687	0.70115	3.00158	0.70124	3.00201
	MSE	0.00259	0.00811	0.00296	0.01776	0.00251	0.00793	0.00268	0.00671
	T.D	0.00327		0.01781		0.00217		0.00243	
500	Mean	0.70030	2.99999	0.70038	3.00269	0.70049	3.00171	0.70019	3.00121
	MSE	0.00121	0.00408	0.00144	0.00851	0.00126	0.00400	0.00133	0.00327
	T.D	0.00042		0.00851		0.00128		0.00067	

**Table 3:** Comparison of the estimation methods for (α = 0.9, θ = 5).

n		${\hat{\alpha }}_{LM}$	${\hat{\theta }}_{LM}$	${\hat{\alpha }}_{TLM}$	${\hat{\theta }}_{TLM}$	${\hat{\alpha }}_{PWM}$	${\hat{\theta }}_{PWM}$	${\hat{\alpha }}_{GPWM}$	${\hat{\theta }}_{GPWM}$
10	Mean	0.93823	5.13723	0.98192	5.26256	0.93675	5.15027	0.91941	5.11158
	MSE	0.15876	0.57927	0.26749	1.61429	0.15478	0.60630	0.12099	0.44208
	T.D	0.06993		0.14353		0.07089		0.04388	
25	Mean	0.91092	5.04928	0.91687	5.08345	0.91004	5.04835	0.90874	5.05122
	MSE	0.04695	0.18460	0.05998	0.39833	0.04605	0.18454	0.04420	0.14228
	T.D	0.02199		0.03544		0.02082		0.01995	
50	Mean	0.90484	5.02623	0.91066	5.03618	0.90564	5.02239	0.89972	5.02691
	MSE	0.02164	0.08289	0.02621	0.17282	0.02141	0.08247	0.02064	0.06723
	T.D	0.01063		0.01908		0.01074		0.00507	
100	Mean	0.90284	5.00993	0.90475	5.01530	0.90198	5.01544	0.90064	5.01154
	MSE	0.01012	0.03879	0.01232	0.08128	0.01032	0.04105	0.01048	0.03199
	T.D	0.00514		0.00833		0.00529		0.00302	
150	Mean	0.90106	5.00664	0.90294	5.00881	0.90196	5.00621	0.89962	5.00980
	MSE	0.00661	0.02684	0.00814	0.05599	0.00676	0.02680	0.00708	0.02126
	T.D	0.00250		0.00503		0.00342		0.00153	
250	Mean	0.90050	5.00348	0.90037	5.00845	0.90014	5.00531	0.90042	5.00569
	MSE	0.00403	0.01515	0.00476	0.03186	0.00397	0.01578	0.00421	0.01238
	T.D	0.00125		0.00210		0.00122		0.00160	
500	Mean	0.90077	5.00247	0.90023	5.00331	0.90046	5.00218	0.90011	5.00321
	MSE	0.00195	0.00783	0.00234	0.01581	0.00202	0.00780	0.00213	0.00614
	T.D	0.00135		0.00092		0.00095		0.00076	

**Table 4:** Comparison of the estimation methods for (α = 2, θ = 1).

n		${\hat{\alpha }}_{LM}$	${\hat{\theta }}_{LM}$	${\hat{\alpha }}_{TLM}$	${\hat{\theta }}_{TLM}$	${\hat{\alpha }}_{PWM}$	${\hat{\theta }}_{PWM}$	${\hat{\alpha }}_{GPWM}$	${\hat{\theta }}_{GPWM}$
10	Mean	2.19286	1.00917	2.29136	1.01476	2.17680	1.00898	1.90477	1.03939
	MSE	0.91882	0.00668	1.39339	0.01403	0.88814	0.00644	0.43085	0.00629
	T.D	0.10560		0.16044		0.09738		0.00823	
25	Mean	2.05348	1.00314	2.08292	1.00417	2.06034	1.00248	1.94897	1.01678
	MSE	0.22583	0.00212	0.29108	0.00386	0.23642	0.00211	0.18125	0.00186
	T.D	0.02988		0.04563		0.03265		0.00873	
50	Mean	2.02212	1.00181	2.03330	1.00335	2.02675	1.00126	1.97213	1.00823
	MSE	0.10626	0.00102	0.12468	0.00173	0.10579	0.00102	0.09320	0.00078
	T.D	0.01287		0.02000		0.01463		0.00571	
100	Mean	2.00907	1.00071	2.01471	1.00129	2.01594	1.00128	1.98011	1.00451
	MSE	0.04898	0.00048	0.05799	0.00084	0.05091	0.00048	0.04704	0.00037
	T.D	0.00524		0.00864		0.00925		0.00544	
150	Mean	2.01030	1.00045	2.01430	1.00074	2.00690	1.00059	1.99235	1.00285
	MSE	0.03232	0.00033	0.03868	0.00056	0.03282	0.00031	0.03117	0.00023
	T.D	0.00560		0.00789		0.00404		0.00098	
250	Mean	2.00772	1.00023	2.00506	1.00069	2.00525	1.00044	1.99295	1.00193
	MSE	0.01949	0.00019	0.02307	0.00033	0.01930	0.00019	0.01900	0.00013
	T.D	0.00409		0.00322		0.00306		0.00159	
500	Mean	2.00311	1.00021	2.00401	1.00007	2.00201	0.99999	1.99660	1.00093
	MSE	0.00950	0.00010	0.01115	0.00016	0.00934	0.00009	0.00937	0.00007
	T.D	0.00176		0.00207		0.00100		0.00077	

As the sample size increases, the estimates of *α* and *θ* generally approach to their true values. The bias is negligible for larger sample sizes, but it is slightly more for smaller sample sizes. Overall, the bias decreases as the sample size increases for all the parameter settings.

Therefore, all the methods show identical performance for estimating the shape and scale parameters of Power function distribution unless the sample size is small. However, the generalized probability weighted moments performs better for smaller sample sizes than other roubust methods considered here such as, L-moments, trimmed L-moments, and probability weighted moments.

### Application

We also compare all the estimation methods on a real data–device failure times. The data set refers to failure times of 30 devices given by Meeker and Escobar [33]. The data are: 275, 13, 147, 23, 181, 30, 65, 10, 300, 173, 106, 300, 300, 212, 300, 300, 300, 2, 261, 293, 88, 247, 28, 143, 300, 23, 300, 80, 245, and 266.

**Fig 1:**
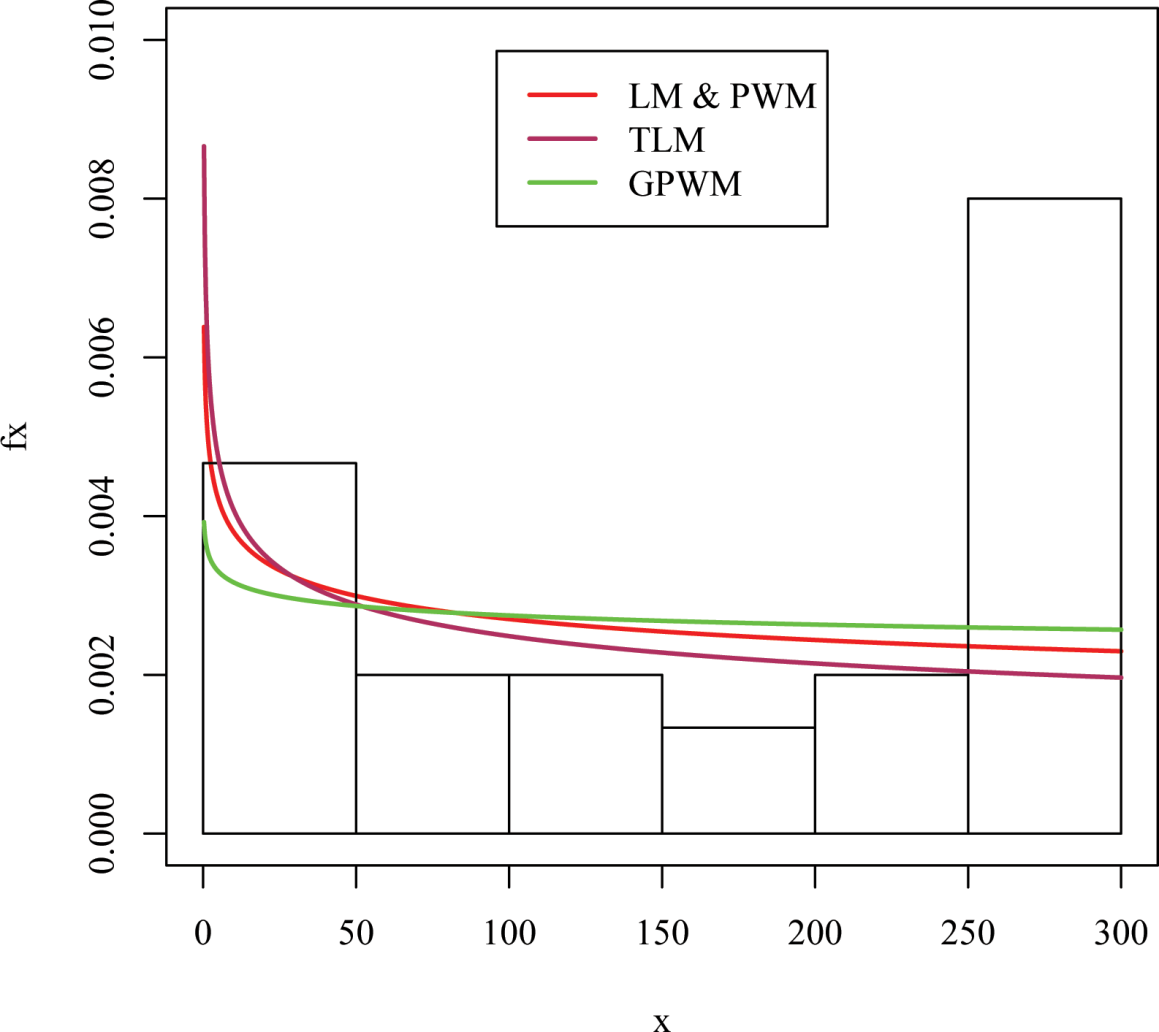
Estimated fitted densities for real data set.

Table 5 shows the estimators of the shape and scale parameters of the power function distribution with KS and AD test statistic along p-values on devices failure time real data. The GPWM is relatively better than other methods in terms of both KS and AD test. Furthermore, Fig 1 shows the appropriateness of the model for failure times data on the basis of histogram and density plots. The plot also confirms that GPWM provides better fit because its curve is relatively close to the empirical density curve.

**Table 5:** Kolmogorov-Smirnov (KS) and Anderson-Darling (AD) test on real data set.

Method	$\hat{\alpha }$	$\hat{\theta }$	KS	AD
L-moments	0.852064	384.804	0.1911 (0.2231)	1.2003 (0.2671)
Trimmed L-moments	0.785252	432.257	0.2493 (0.0479)	1.3900 (0.21)
Probability Weighted Moments	0.852064	384.804	0.1911 (0.2231)	1.2003 (0.2671)
Generalized Probability Weighted Moments	0.938654	370.1152	0.1789 (0.2920)	1.3241 (0.2245)

*Note*: P-values of the KS test statistic and AD test statistic are given in parentheses.

## Conclusion

In this study, we introduce two new parameter estimation methods such as, probability weighted moments and generalized probability moments for power function distribution. It is a flexible and simple distribution that may helpful for modeling the failure data. Mathematical expressions of the estimators are derived for the L-moments, TL-moments, PWM, and GPWM. We compare the performance of these methods for power function distribution through a simulation study and read data. Therefore, it is concluded from both simulated and real data that all the methods show identical performance for estimating the shape and scale parameters of Power function distribution unless the sample size is small. However, the generalized probability weighted moments performs better for smaller sample sizes than other roubust methods considered here such as, L-moments, trimmed L-moments, and probability weighted moments.
