# Correction: Comparison of Two New Robust Parameter Estimation Methods for the Power Function Distribution

**DOI:** 10.1371/journal.pone.0162536

**Published:** 2016-09-01

**Authors:** Muhammad Shakeel, Muhammad Ahsan ul Haq, Ijaz Hussain, Alaa Mohamd Abdulhamid, Muhammad Faisal

An affiliation is missing for the fifth author, Muhammad Faisal. Muhammad Faisal is affiliated with: Bradford Institute for Health Research, Bradford Teaching Hospitals, NHS Foundation Trust, Bradford, UK and with the Faculty of Health Studies, University of Bradford, BD7 1DP Bradford, UK.
